# OMERACT-based fibromyalgia symptom subgroups: an exploratory cluster analysis

**DOI:** 10.1186/s13075-014-0463-7

**Published:** 2014-10-16

**Authors:** Ann Vincent, Tanya L Hoskin, Mary O Whipple, Daniel J Clauw, Debra L Barton, Roberto P Benzo, David A Williams

**Affiliations:** Division of General Internal Medicine, Mayo Clinic, 200 First Street SW, Rochester, MN 55902 USA; Division of Biomedical Statistics and Informatics, Mayo Clinic, 200 First Street SW, Rochester, MN 55902 USA; Chronic Pain and Fatigue Research Center, University of Michigan Health System, 24 Frank Lloyd Wright Drive, Ann Arbor, MI 48105 USA; Division of Medical Oncology, Mayo Clinic, 200 First Street SW, Rochester, MN 55902 USA; Division of Pulmonary and Critical Care Medicine, Mayo Clinic, 200 First Street SW, Rochester, MN 55902 USA

## Abstract

**Introduction:**

The aim of this study was to identify subsets of patients with fibromyalgia with similar symptom profiles using the Outcome Measures in Rheumatology (OMERACT) core symptom domains.

**Methods:**

Female patients with a diagnosis of fibromyalgia and currently meeting fibromyalgia research survey criteria completed the Brief Pain Inventory, the 30-item Profile of Mood States, the Medical Outcomes Sleep Scale, the Multidimensional Fatigue Inventory, the Multiple Ability Self-Report Questionnaire, the Fibromyalgia Impact Questionnaire–Revised (FIQ-R) and the Short Form-36 between 1 June 2011 and 31 October 2011. Hierarchical agglomerative clustering was used to identify subgroups of patients with similar symptom profiles. To validate the results from this sample, hierarchical agglomerative clustering was repeated in an external sample of female patients with fibromyalgia with similar inclusion criteria.

**Results:**

A total of 581 females with a mean age of 55.1 (range, 20.1 to 90.2) years were included. A four-cluster solution best fit the data, and each clustering variable differed significantly (*P* <0.0001) among the four clusters. The four clusters divided the sample into severity levels: Cluster 1 reflects the lowest average levels across all symptoms, and cluster 4 reflects the highest average levels. Clusters 2 and 3 capture moderate symptoms levels. Clusters 2 and 3 differed mainly in profiles of anxiety and depression, with Cluster 2 having lower levels of depression and anxiety than Cluster 3, despite higher levels of pain. The results of the cluster analysis of the external sample (*n* = 478) looked very similar to those found in the original cluster analysis, except for a slight difference in sleep problems. This was despite having patients in the validation sample who were significantly younger (*P* <0.0001) and had more severe symptoms (higher FIQ-R total scores (*P* = 0.0004)).

**Conclusions:**

In our study, we incorporated core OMERACT symptom domains, which allowed for clustering based on a comprehensive symptom profile. Although our exploratory cluster solution needs confirmation in a longitudinal study, this approach could provide a rationale to support the study of individualized clinical evaluation and intervention.

## Introduction

Despite chronic, widespread pain being its cardinal symptom, fibromyalgia (FM) actually presents as a heterogeneous symptom complex that remains poorly understood [1,2]. In addition to pain, frequently occurring symptoms include fatigue, unrefreshing sleep, cognitive difficulties, headache, anxiety and depression [3]. Patients with FM often present with varying combinations and degrees of severity of these symptoms, which further complicates the ability to understand FM and makes its study challenging [4]. Stratifying patients into more homogeneous subgroups may facilitate better understanding of FM.

Several studies have reported homogeneous subgroups within samples of patients with FM discovered using the exploratory data-mining technique of cluster analysis [5-11]. Variables used to identify clusters in these studies have included sociodemographic variables, patient responses to self-report questionnaires, physiological and psychophysiological parameters, biomarkers, comorbidities and psychosocial functioning. For example, Giesecke *et al*. categorized patients with FM based on self-report measures and evoked pain testing [6]. In their study, 97 patients were clustered into three subgroups: (1) extreme tenderness with normal mood, (2) low tenderness with moderate mood and (3) extreme tenderness with low mood. In another study, by de Souza *et al*., patients with FM were clustered into two groups based on their responses to the Fibromyalgia Impact Questionnaire (FIQ) [5]. In that study, both clusters reported similar levels of pain, fatigue and stiffness, but differed on severity of morning tiredness, anxiety and depression. Similarly, Docampo *et al*. clustered a large sample of Spanish patients with FM into three groups using dimensions of symptomatology, comorbidities and clinical scales. These subgroups included (1) low symptomatology and comorbidities, (2) high symptomatology and comorbidities and (3) high symptomatology and low comorbidities. These studies illustrate the symptom spectrum and symptom combinations that are possible in a heterogeneous sample of patients carrying the same diagnosis, and the results are consistent with those of Loevinger *et al*. and Wilson *et al*. [7,10]. It has also been reported that patients with FM belonging to different clusters may respond differently to pain rehabilitation. In separate studies, Verra *et al*. and Turk *et al*. demonstrated that (1) the same treatment can yield different responses, depending upon symptom constitution of a subgroup; and (2) tailoring treatment based on symptoms could improve treatment efficacy [9,12].

Although a wide range of variables have been used to identify clusters of patients with FM, few studies have included a comprehensive symptom profile. The Outcome Measures in Rheumatology (OMERACT) FM working group recommended 12 domains (including both symptoms and biomarkers) to be used in FM studies: pain, fatigue, sleep disturbance, depression, anxiety, stiffness, dyscognition, patient global impression of health, multidimensional functioning, tenderness, cerebrospinal fluid (CSF) biomarkers and pain-related neuroimaging markers (if available) [13]. The OMERACT domains provide an all-inclusive list of symptoms that may be used to better understand FM. Our objective in the present study was to identify clusters within a heterogeneous sample of patients with FM using OMERACT symptom domains assessed with validated, self-report questionnaires. We then sought to validate our cluster results in an independent sample of patients with FM.

## Methods

### Ethics statement

The study was approved by the Mayo Clinic Institutional Review Board, and all participants provided written informed consent.

Data for the exploratory cluster analysis were derived from survey responses of a sample of patients randomly selected from an existing FM registry that has been described previously [14]. Briefly, the FM registry is composed of patients who have been seen at the Mayo Clinic with a diagnosis of FM since 1 January 2000 and have consented to enrollment in the registry. Participants were invited to participate by postal survey and completed a questionnaire package that included the Brief Pain Inventory (BPI) [15], the 30-item Profile of Mood States (30-item POMS) [16], the Medical Outcomes Study Sleep Scale (MOS-Sleep) [17], the Multidimensional Fatigue Inventory (MFI-20) [18], the Multiple Ability Self-report Questionnaire (MASQ) [19], the Revised Fibromyalgia Impact Questionnaire (FIQ-R) [20] and the 36-item Short Form survey (SF-36) [21]. All instruments included are considered appropriate in meaningfully characterizing the multiple facets of FM as described by Williams and Arnold [22].

### Participants

Only female respondents who met FM research survey criteria were included in the exploratory cluster analysis [23]. Male respondents were excluded (*n* = 50), because the number of males in our sample was too small to analyze meaningfully, given evidence which suggests that the symptom experience for males with FM may differ from that of females [24-27].

### Measures

OMERACT-recommended symptom domains were assessed using either total scores or subscale scores of the questionnaires described below [28]. Our goal was to select the scale or subscale that best represented the OMERACT symptom domain while eliminating the possibility of item overlap between symptom domains and clustering variables. For example, in the case of MFI-20, which includes physical fatigue, mental fatigue (related to cognition) and reduced motivation (related to mood), we purposefully selected physical fatigue to keep this symptom construct distinct. The subscales selected for each domain are shown in Table 1. Of the core OMERACT symptom domains, all except objective tenderness and patient global impression of change to treatment (which was not applicable, given that there was no treatment comparison) were available. Table 1 lists the measures used to operationalize OMERACT domains (that is, used for clustering) and those measures used as descriptive clinical outcomes against which the clusters were evaluated.

**Table 1:** **Scales and subscales representing OMERACT domains** ^**a**^

**OMERACT domain**	**Scale/Subscale selected**
Pain	BPI Pain Severity
Fatigue	MFI Physical Fatigue
Function	FIQ Function
Sleep disturbance	MOS Sleep Problems Index II
Depression	POMS Depression Dejection
Dyscognition	MASQ Total
Anxiety	POMS Tension Anxiety
Stiffness	FIQ Stiffness
Tenderness	NA
Patient global assessment	NA
CSF biomarkers	NA
Functional Imaging	NA

^a^BPI, Brief Pain Inventory; CSF, Cerebrospinal fluid; FIQ, Fibromyalgia Impact Questionnaire; MASQ, Multiple Ability Self-report Questionnaire; MFI, Multidimensional Fatigue Inventory; MOS-Sleep, Medical Outcomes Study Sleep Scale; NA, Not applicable; OMERACT, Outcome Measures in Rheumatology; POMS, Profile of Mood States.

#### Pain (Brief Pain Inventory)

The BPI is a 15-item, validated self-report measure of chronic, non-cancer-related pain and assesses presence of pain, pain severity and pain interference. It yields two subscales: pain severity and pain interference. Scores on pain severity and pain interference range from 0 to 10, with higher scores indicating greater pain. Internal consistency for the Pain Severity score is 0.85 and that for the Interference scale is 0.88 [29]. The BPI has been used in FM clinical trials and is considered an appropriate measure of pain in FM [22,30-33]. For this analysis, we selected the Pain Severity subscale to represent the OMERACT symptom domain of pain.

#### Depression and anxiety (30-item Profile of Mood States)

The 30-item POMS is a validated self-report measure of mood. It yields six subscales: (1) depression-dejection, (2) tension-anxiety, (3) fatigue-inertia, (4) vigor-activity, (5) anger-hostility and (6) confusion-bewilderment. Scores on each subscale range from 0 to 20, with higher scores indicating worse symptoms on all scales, except for the vigor-activity scale, on which lower scores indicate worse symptoms. Although the majority of publications describing POMS in FM have been based on the 65-item instrument, the 30-item POMS has an internal consistency of 0.69 to 0.88 and is considered a superior measure in general [34-36]. For this analysis, we selected the Depression-Dejection and Tension-Anxiety subscales to represent the OMERACT symptom domains of depression and anxiety.

#### Sleep disturbance (Medical Outcomes Study-Sleep scale)

The MOS-Sleep scale is a 12-item, validated, self-report measure that assesses six dimensions of sleep: (1) sleep disturbance, (2) sleep adequacy, (3) sleep quantity, (4) somnolence, (5) snoring and (6) awakening with shortness of breath or headache. It yields two summary indices: the Sleep Problems Index I (six items) and the Sleep Problems Index II (nine items). Scores on dimensions and summary indices range from 0 to 100, with higher scores indicating poorer sleep. The MOS-Sleep scale has an internal consistency of 0.7 in patients with FM [17]. The MOS-Sleep scale has been used in many FM clinical trials and is considered an appropriate measure of sleep in FM [37-39]. For this analysis, we selected the Sleep Problems Index II to represent the OMERACT symptom domain of sleep disturbance.

#### Fatigue (Multidimensional Fatigue Inventory)

The MFI-20 is a 20-item validated self-report measure of fatigue and assesses general fatigue, physical fatigue, reduced activity, reduced motivation and mental fatigue [18]. Subscale scores range from 4 to 20, with higher scores indicating greater fatigue. It has an internal consistency of 0.93 [40]. The MFI-20 has been used in clinical trials of FM and chronic pain and is an appropriate measure of fatigue in FM [31,41,42]. For this analysis, we selected the MFI Physical Fatigue subscale to represent the OMERACT symptom domain of fatigue.

#### Dyscognition (Multiple Ability Self-report Questionnaire)

The MASQ is a 38-item self-report measure and assesses five cognitive domains: language, visuoperceptual, verbal memory, visual memory and attention [19]. Scores on the cognitive domains range from 0 to 30 or 0 to 40, and the maximum total score is 190. Higher scores indicate greater perceived difficulties with cognition. The MASQ has an internal consistency of 0.92. It has been used in several FM clinical trials to measure change in perceived cognition [43-45]. For this analysis, we selected the MASQ total to represent the OMERACT symptom domain of dyscognition.

#### Stiffness (Revised Fibromyalgia Impact Questionnaire)

The FIQ-R is a 21-item, validated self-report measure that assesses the symptoms, physical functioning and overall impact of FM [20]. Scores range from 0 to 100, with higher scores indicating greater symptom burden. It has an internal consistency of 0.95 and is the most commonly used outcome measure in FM clinical trials [22,37,46,47]. For this analysis, we selected the FIQ-R Stiffness question to represent the OMERACT symptom domain of stiffness. Additionally, FIQ-R total scores (not used in the clustering) were compared across the subgroups identified by cluster analysis.

#### SF-36

The SF-36 version 2 is a 36-item, validated self-report measure that assesses disease burden [21]. It consists of eight subscales and two summary scores (physical and mental components). Component scores range from 0 to 100, with higher scores indicating better health. The SF-36 has an internal consistency of 0.9 and has been used in FM clinical trials [48-50]. Similarly to the FIQ-R total score, the SF-36 component scores were not included in the clustering, but were used for comparison of symptom levels across clusters.

#### Fibromyalgia research survey criteria

The FM research survey criteria have been validated for use in epidemiologic and survey studies [23]. It yields a widespread pain index (WPI) score (range, 0 to 19) and a symptom severity (SS) score of (0 to 12). Patients are classified as meeting FM research survey criteria if their WPI was ≥7 and SS was ≥5 or if their WPI was between 3 and 6 SS ≥9. Higher scores indicate more severe symptoms. WPI and SS scores were also compared across clusters.

### Participants and measures in the external validation sample

To provide an external validation measure, we chose a separate sample of patients with FM (*n* = 478) who participated in the FM Treatment Program at Mayo Clinic but were not yet enrolled in the FM registry. Patients who participate in this program complete a comprehensive package of questionnaires. This comprehensive package includes the same measures used in the exploratory cluster analysis, with the exception of POMS and BPI. In this sample, in place of POMS, the severity of depression and anxiety were assessed with Patient Health Questionnaire (PHQ-9) and Generalized Anxiety Disorder 7-item scale (GAD-7), both of which are clinically validated measures [51-53]. Although BPI was not part this package, the Pain subscale of the FIQ provides a similar measure of pain severity and is considered appropriate to meaningfully characterize pain in FM, as described by Williams and Arnold [22]. Similarly to the exploratory cluster sample, only female participants who met FM Research Survey Criteria were included. Importantly, medication use at the time of the survey (that is, the clinic visit) was available for participants included in the validation sample, whereas such data were not available in the original registry survey–based cluster sample.

### Statistical methods

Cluster analysis variables were standardized by subtracting the variable mean from each individual observation and dividing by the standard deviation. Hierarchical agglomerative clustering with Ward’s method and squared Euclidean distances was used on the standardized data. The number of clusters was chosen by examining the dendrogram and based on clinical interpretability and usefulness. Clusters were subsequently compared on variables of interest using analysis of variance, followed by pairwise comparisons between clusters when the omnibus test was significant (that is, *P* <0.05). For pairwise comparisons, a Bonferroni adjustment to the 0.05 significance level was applied when interpreting *P*-values; because our analysis resulted in four clusters, and thus six pairwise comparisons, the Bonferroni *P*-value for interpreting pairwise significance tests was 0.05/6 = 0.0083. Analysis was performed using R [54] statistical software (version 2.15.0; R Foundation for Statistical Computing, Vienna, Austria) and JMP (version 10.0.0; SAS Institute, Cary, NC, USA). The same methods used for cluster analysis and comparison across clusters were applied to the validation sample. Because standardized variables (that is, z-scores) rather than raw scales were used for the cluster analysis, we felt it was reasonable to interpret and compare results from the validation and original samples, although a few of the specific instruments to measure individual symptoms differed between the two samples, as described above.

## Results

### Exploratory cluster analysis

Of the 655 patients who met the inclusion criteria for the exploratory cluster analysis, 74 patients had missing data for one or more of the cluster analysis variables, yielding a final sample size of 581 patients for analysis. Demographic and symptom scales for the sample are summarized in Table 2.

**Table 2:** **Patient characteristics of 581 patients included in cluster analysis** ^**a**^

**Characteristics**	**Mean (SD) or** ***n*** **(%)**	**Median (range)**
Age, yr	55.1 (12.4)	55.6 (20.1 to 90.2)
Race		
White	531 (91.4)	
Other	10 (1.7)	
Missing data	40 (6.9)	
Education		
Less than high school	13 (2.2)	
High school graduate or GED	122 (21.0)	
Some college or 2-year degree	225 (38.7)	
4-year college graduate	92 (15.8)	
Postgraduate studies	86 (14.8)	
Missing data	43 (7.4)	
Employment status		
Employed	206 (35.4)	
Retired	124 (21.3)	
Work-disabled	109 (18.8)	
Other (student, full-time homemaker, other)	69 (11.9)	
Unemployed	51 (8.8)	
Missing data	22 (3.8)	
Marital status		
Married/committed relationship	435 (74.9)	
Divorced/separated	55 (9.4)	
Single	44 (7.6)	
Widowed	25 (4.3)	
Missing data	22 (3.8)	
BMI, kg/m^2^	30.1 (7.6)	29.2 (12.6 to 59.2)
FM research survey		
WPI	12.6 (3.8)	13 (3 to 19)
SS	8.9 (2.0)	9 (5 to 12)
BPI		
Pain severity	5.1 (1.8)	5.3 (0.3 to 10)
Pain interference	5.7 (2.3)	5.9 (0 to 16.3)
MOS-Sleep Problems Index II	55.1 (18.9)	56.7 (8.9 to 97.8)
POMS		
Depression-Dejection	6.8 (5.1)	6 (0 to 20)
Tension-Anxiety	7.2 (4.8)	7 (0 to 20)
MFI-20		
General	17.3 (2.6)	18 (7 to 20)
Physical	15.8 (3.6)	17 (4 to 20)
Reduced activity	14.6 (3.9)	15 (4 to 20)
Reduced motivation	12.4 (3.8)	12 (4 to 20)
Mental	13.2 (4.2)	13 (4 to 20)
Total	73.3 (14.0)	74 (29 to 100)
MASQ	95.4 (22.0)	94 (49 to 162)
FIQ-R total	56.0 (18.7)	57.5 (5 to 96.2)
Stiffness	7.2 (2.2)	8 (0 to 10)
SF-36		
Physical	30.3 (8.6)	29.8 (8.3 to 52.9)
Mental	40.2 (12.6)	40.5 (7.5 to 66.8)

^a^BMI, Body mass index; BPI, Brief Pain Inventory; FIQ-R, Revised Fibromyalgia Impact Questionnaire; FM, Fibromyalgia; GED, General educational development; MASQ, Multiple Ability Self-Report Questionnaire ; MFI, Multidimensional Fatigue Inventory; MOS-Sleep, Medical Outcomes Study Sleep Scale; POMS, Profile of Mood States; SF-36, Medical Outcomes Study 36-item Short Form survey; SS, Symptom severity; WPI, Widespread pain index.

**Figure 1:**
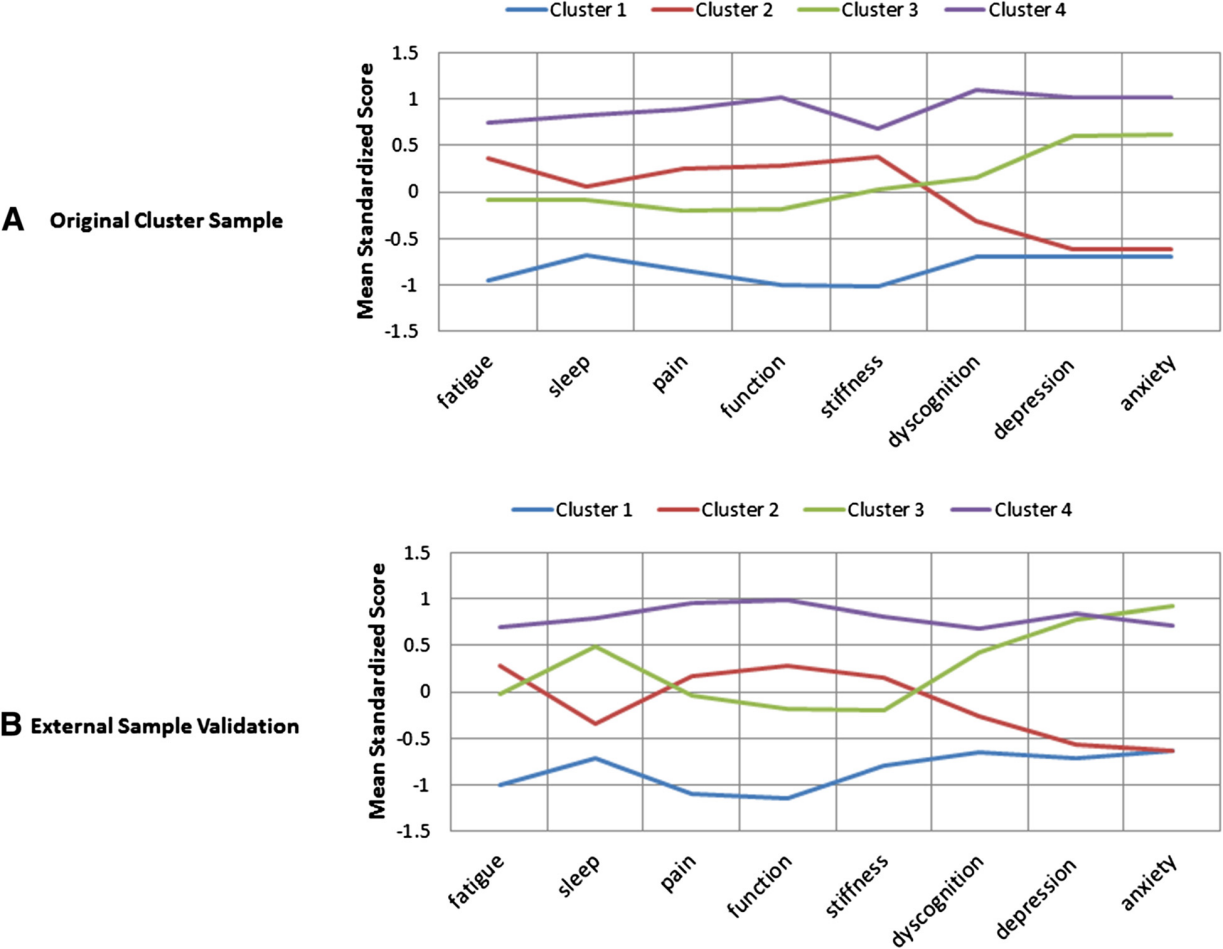
**Cluster profiles plot for each of the two study samples. (A)** Original sample. **(B)** External validation sample.

Hierarchical agglomerative clustering on the standardized variables corresponding to fatigue, sleep, pain, function, stiffness, dyscognition, depression and anxiety resulted in a dendrogram that suggested meaningful information when the data were examined with between three and four clusters. The four-cluster solution largely divided the sample into severity levels, with cluster 1 reflecting the lowest average levels across all symptoms, cluster 4 reflecting the highest average levels across all symptoms and clusters 2 and 3 capturing generally moderate symptom levels. An important distinction between clusters 2 and 3 was their different profiles on the mental aspects of the disease, as cluster 2 clearly had lower levels of depression and anxiety than did cluster 3, despite cluster 2’s having somewhat higher levels of pain, stiffness, dysfunction, sleep disturbance and fatigue (Figure 1A). Considering a three-cluster solution, clusters 1 and 2 would have remained together, which we felt would miss a clinically important difference revealed by the four-cluster solution because these two subgroups have significantly different levels of fatigue, sleep, pain, stiffness, function and dyscognition despite their similar levels of negative mood. Thus, the four-cluster solution was used for the subsequent analyses described below.

### Characterizing clusters

The four clusters were similar in size, with clusters 1 to 4 comprising 26%, 29%, 23% and 21% of the samples, respectively. Each of the clustering variables differed significantly between the four clusters (Table 3). The pairwise comparisons also showed significant differences of each cluster from every other cluster on all symptoms, with a few exceptions. Clusters 2 and 3 did not differ significantly on sleep problems (*P* = 0.16), and clusters 1 and 2 did not differ significantly on either depression (*P* = 0.23) or anxiety (*P* = 0.26).

**Table 3:** **Cluster analysis variables compared across clusters** ^**a**^

**Measure**	**Cluster 1** ^**b**^ **(** ***n*** **= 150)**	**Cluster 2** ^**c**^ **(** ***n*** **= 171)**	**Cluster 3** ^**d**^ **(** ***n*** **= 136)**	**Cluster 4** ^**e**^ **(** ***n*** **= 124)**	***P*** **-value** ^**f**^
MFI physical fatigue	12.5 (3.7)	17.1 (2.4)	15.5 (2.8)	18.5 (1.7)	<0.0001
MOS-Sleep Problems Index II	42.4 (16.5)	56.2 (17.2)	53.5 (15.4)	70.8 (15.5)	<0.0001
BPI pain severity	3.6 (1.4)	5.5 (1.4)	4.7 (1.5)	6.6 (1.2)	<0.0001
MASQ	80.1 (15.7)	88.5 (16.9)	98.8 (17.8)	119.5 (17.4)	<0.0001
FIQ function	7.1 (4.8)	16.2 (5.7)	12.9 (5.2)	21.5 (4.0)	<0.0001
FIQ stiffness	5.0 (2.2)	8.0 (1.4)	7.2 (1.8)	8.7 (1.1)	<0.0001
POMS tension-anxiety	3.9 (3.1)	4.3 (2.6)	10.2 (3.6)	12.1 (3.8)	<0.0001
POMS Depression-Dejection	3.2 (3.2)	3.7 (2.5)	10.0 (3.7)	12.1 (4.5)	<0.0001

^a^Data are mean ± SD. BPI, Brief Pain Inventory; FIQ, Fibromyalgia Impact Questionnaire; MASQ, Multiple Ability Self-report Questionnaire; MFI, Multidimensional Fatigue Inventory; MOS-Sleep Problems Index II, Medical Outcomes Study Sleep scale; POMS, Profile of Mood States; SF-36, Medical Outcomes Study 36-item Short Form survey. ^b^Low symptom intensity. ^c^Moderate symptoms, low negative mood. ^d^Moderate symptoms, higher negative mood and dyscognition. ^e^High symptom intensity. ^f^ *P*-values reported are from the omnibus analysis of variance test across the four clusters. Pairwise comparisons between clusters also showed significant differences in all cases, except for the following: cluster 2 versus cluster 3 MOS-Sleep Problems Index II (*P* = 0.16) and cluster 1 versus cluster 2 with respect to both POMS Tension-Anxiety (*P* = 0.26) and Depression-Dejection (*P* = 0.23).

Clinically relevant variables external to the cluster creation were compared across clusters, and the results are given in Table 4. FIQ Impact was significantly different for each cluster (all *P*-values <0.0001), except for cluster 2 versus cluster 3, which had very similar levels (mean, 11.5 vs 11.7; *P* =0.76). Similarly, clusters 2 and 3 had similar FIQ Total scores (mean, 58.1 vs 57.7). This underscores the point that both clusters have a globally “moderate” level of symptoms, although the drivers appear to be more physical in cluster 2 and more mental in cluster 3. The SF-36 mental composite score differed significantly for each cluster, and the physical composite score differed for every cluster comparison except cluster 2 versus cluster 4 (mean, 26.2 vs 25.6; *P* = 0.53).

**Table 4:** **Nonclustering variables compared across clusters** ^**a**^

**Variables**	**Cluster 1** ^**b**^ **(** ***n*** **= 150)**	**Cluster 2** ^**c**^ **(** ***n*** **= 171)**	**Cluster 3** ^**d**^ **(** ***n*** **= 136)**	**Cluster 4** ^**e**^ **(** ***n*** **= 124)**	***P*** **-value** ^**f**^
Age, yr	57.0 (14.5)	57.6 (11.3)	52.5 (12.4)	52.2 (9.7)	<0.0001
BMI, kg/m^2^	28.6 (7.6)	31.5 (7.8)	29.2 (6.9)	30.8 (7.8)	0.0030
FM research survey					
WPI	11.1 (3.5)	12.8 (3.7)	12.5 (3.6)	14.3 (3.5)	<0.0001
SS	7.6 (1.8)	8.7 (1.8)	9.1 (1.8)	10.5 (1.4)	<0.0001
BPI Pain interference	3.4 (1.9)	5.9 (1.8)	5.9 (1.6)	7.9 (1.3)	<0.0001
MFI-20					
General	15.0 (2.8)	17.8 (2.1)	17.5 (2.2)	19.0 (1.6)	<0.0001
Reduced activity	11.7 (3.9)	15.0 (3.4)	14.6 (3.4)	17.6 (2.3)	<0.0001
Reduced motivation	9.8 (3.1)	12.1 (3.5)	12.9 (3.3)	15.5 (2.9)	<0.0001
Mental	10.5 (3.8)	12.4 (3.8)	13.9 (3.9)	16.6 (2.9)	<0.0001
Total	59.3 (11.8)	74.5 (10.5)	74.4 (10.4)	87.1 (7.1)	<0.0001
SF-36					
Physical^g^	36.5 (8.3)	26.2 (7.3)	33.1 (6.8)	25.6 (6.4)	<0.0001
Mental^g^	48.9 (9.3)	45.4 (10.1)	34.5 (9.3)	28.6 (10.3)	<0.0001
FIQ					
Tenderness	5.8 (2.8)	7.8 (2.3)	7.7 (2.3)	8.8 (1.6)	<0.0001
Impact	5.7 (4.5)	11.5 (5.1)	11.7 (4.7)	16.3 (3.7)	<0.0001
Total	34.3 (11.7)	58.1 (13.0)	57.7 (10.8)	77.7 (8.6)	<0.0001
POMS					
Anger-Hostility	3.0 (3.3)	3.2 (2.7)	6.8 (4.2)	8.6 (4.4)	<0.0001
Vigor-Activity^g^	6.5 (3.6)	3.9 (3.1)	3.8 (2.8)	2.4 (2.6)	<0.0001
Fatigue-Inertia	10.2 (3.7)	13.6 (3.6)	14.7 (3.1)	16.9 (2.6)	<0.0001
Confusion-Bewilderment	4.7 (2.9)	6.1 (2.7)	9.1 (2.8)	12.7 (3.2)	<0.0001

^a^Data are mean ± SD. BMI, Body mass index; BPI, Brief Pain Inventory; FIQ, Fibromyalgia Impact Questionnaire; FM, Fibromyalgia; MFI, Multidimensional Fatigue Inventory; POMS, Profile of Mood States; SF-36, 36-item Medical Outcomes Study Short Form health survey; SS, Symptom severity; WPI, Widespread pain index. ^b^Low symptom intensity. ^c^Moderate symptoms, low negative mood. ^d^Moderate symptoms, higher negative mood and dyscognition. ^e^High symptom severity. ^f^ *P*-values are from the omnibus analysis of variance test across the four clusters. Pairwise comparisons between clusters also showed significant differences in all cases, except for the following: cluster 1 versus cluster 2 with respect to age (*P* = 0.62), cluster 1 versus cluster 3 with respect to BMI (*P* = 0.51), cluster 2 versus cluster 4 with respect to both BMI (*P* = 0.49) and SF-36 Physical (*P* = 0.53) and cluster 3 versus cluster 4 with respect to both age (*P* = 0.86) and BMI (*P* = 0.08). Differences between clusters 2 and 3 were not significant for all variables, except age, BMI, MFI Reduced Motivation, MFI Mental Fatigue, SF-36 Physical, SF-36 Mental, POMS Fatigue-Inertia and POMS Confusion-Bewilderment. ^g^Lower scores are worse for these scales.

Interestingly, patients in clusters 1 and 2, who had the lowest levels of depression and anxiety, were also significantly older than those in clusters 3 and 4. Body mass index (BMI) varied somewhat across clusters, but the only pairwise comparison that reached statistical significance was for cluster 1 versus 2 (mean, 28.6 vs 31.5; *P* = 0.0008).

### External sample validation

A total of 478 female patients who met the FM research survey criteria were included in the validation sample. The mean age of the participants was 46.5 (±13.1) years, mean BMI was 30.2 (±7.8) kg/m^2^ and the mean FIQ Total score was 60.0 (±17.4). Compared to the original sample, the validation sample was significantly younger (*P* <0.0001), had a similar BMI (*P* = 0.74) and had a higher FIQ Total score (*P* = 0.0004).

As with the exploratory cluster analysis, the dendrogram of the hierarchical clustering solution for the validation sample suggested four clusters. The four clusters comprised 24%, 32%, 21% and 23% of the sample, respectively. The four-cluster solution from the validation sample similarly divided the sample into severity levels, with cluster 1 demonstrating the lowest symptom severity and cluster 4 having the highest symptom severity (Figure 1B). Similarly to the exploratory cluster analysis, each of the clustering variables differed significantly among the four clusters (Table 5). The pairwise comparisons also showed significant differences of each cluster from every other cluster on all symptoms, with a few exceptions. Clusters 1 and 2 did not differ significantly on anxiety (*P* = 0.96) or depression (*P* = 0.07). Clusters 3 and 4 did not differ significantly on depression (*P* = 0.53). The symptom profiles looked very similar to those found in the original cluster analysis sample, with a slight difference for sleep problems, which did not differ significantly between clusters 2 and 3 in the original sample but were significantly worse for cluster 3 vs cluster 2 in the validation sample.

**Table 5:** **Cluster analysis variables compared across clusters in the validation sample** ^**a**^

**Measure**	**Cluster 1** ^**b**^ **(** ***n*** **= 116)**	**Cluster 2** ^**c**^ **(** ***n*** **= 154)**	**Cluster 3** ^**d**^ **(** ***n*** **= 100)**	**Cluster 4** ^**e**^ **(** ***n*** **= 108)**	***P*** **-value** ^**f**^
MFI physical fatigue	13.0 (3.9)	17.5 (2.3)	16.4 (2.7)	18.9 (1.8)	<0.0001
MOS-Sleep problems Index II	46.1 (15.9)	52.9 (16.3)	68.5 (14.8)	74.1 (12.3)	<0.0001
FIQ pain	4.8 (1.5)	7.1 (1.3)	6.7 (1.2)	8.5 (0.9)	<0.0001
MASQ	82.7 (18.1)	91.3 (21.3)	107.0 (17.1)	112.8 (19.5)	<0.0001
FIQ function	7.0 (4.8)	17.4 (5.2)	14.0 (5.2)	22.6 (3.5)	<0.0001
FIQ stiffness	5.4 (2.2)	7.5 (1.8)	6.7 (2.2)	8.9 (1.0)	<0.0001
GAD-7	5.0 (4.1)	5.0 (3.7)	14.2 (4.0)	13.0 (4.7)	<0.0001
PHQ-9	8.6 (4.1)	9.5 (3.2)	17.1 (4.5)	17.4 (4.3)	<0.0001

^a^ *N* = 478 total validation sample. Data are *n* (%). FIQ, Revised Fibromyalgia Impact Questionnaire; GAD-7, Generalized Anxiety Disorder 7-item scale; MASQ, Multiple Ability Self-report Questionnaire; MFI, Multidimensional Fatigue Inventory; MOS-Sleep Problems Index II, Medical Outcomes Study Sleep Scale; PHQ-9, Patient Health Questionnaire. ^b^Low symptom intensity. ^c^Moderate symptoms, low negative mood. ^d^Moderate symptoms, higher negative mood and dyscognition. ^e^High symptom intensity. ^f^ *P*-values are from the omnibus analysis of variance test across the four clusters. Pairwise comparisons between clusters also showed significant differences in all cases, except for the following: cluster 1 versus cluster 2 in regard to GAD-7 (*P* = 0.96) and PHQ-9 (*P* = 0.07) and cluster 3 versus cluster 4 with respect to PHQ-9 (*P* = 0.53).

The FIQ-Total and SF-36 scores serve as external benchmarks for the cluster differentiation. As with the original cluster sample, these scores are consistent with our interpretation of the clusters, specifically that cluster 1 shows a globally low symptom level, cluster 4 shows a globally high symptom level and clusters 2 and 3 are both generally moderate at the global level based on FIQ-Total scores (Table 6), whereas the SF-36 Mental component score differentiated clusters 2 and 3.

**Table 6:** **Nonclustering variables, including medication use, compared across clusters in external sample** ^**a**^

**Variables**	**Cluster 1** ^**b**^ **(** ***n*** **= 116)**	**Cluster 2** ^**c**^ **(** ***n*** **= 154)**	**Cluster 3** ^**d**^ **(** ***n*** **= 100)**	**Cluster 4** ^**e**^ **(** ***n*** **= 108)**	***P*** **-value** ^**f**^
Age, yr	49.3 (15.2)	47.4 (13.2)	45.1 (12.1)	43.7 (10.9)	0.0074
BMI, kg/m^2^	29.1 (6.7)	31.3 (8.5)	29.7 (8.3)	30.3 (7.2)	0.14
FM research survey					
WPI	12.1 (3.6)	13.2 (3.7)	12.9 (3.6)	14.3 (3.3)	<0.0001
SS	7.1 (1.7)	8.1 (1.7)	9.3 (1.5)	9.6 (1.5)	<0.0001
SF-36					
Physical^g^	36.3 (7.4)	26.2 (7.0)	31.7 (6.8)	24.6 (5.7)	<0.0001
Mental^g^	45.0 (10.6)	42.6 (10.2)	29.7 (9.8)	31.6 (9.8)	<0.0001
FIQ total	37.9 (10.7)	61.3 (10.8)	62.4 (9.2)	79.3 (7.9)	<0.0001
Medications					
SNRI	26 (22.4)	37 (24.0)	34 (34.0)	48 (44.4)	0.0007
TCA	12 (10.3)	20 (13.0)	14 (14.0)	10 (9.3)	0.66
α-2-δ ligand	23 (19.8)	44 (28.6)	24 (24.0)	32 (29.6)	0.29
SSRI	22 (19.0)	36 (23.4)	29 (29.0)	26 (24.1)	0.39
Others^h^	17 (14.7)	18 (11.7)	23 (23.0)	18 (16.7)	0.11
Any medication that could be used to treat depression (above)	72 (62.1)	105 (68.2)	76 (76.0)	83 (76.9)	0.0488
Tramadol	12 (10.3)	22 (14.3)	15 (15.0)	20 (18.5)	0.38
Skeletal muscle relaxants	20 (17.2)	43 (27.9)	24 (24.0)	33 (30.6)	0.10
Opioids	21 (18.1)	51 (33.1)	28 (28.0)	57 (52.8)	<0.0001
Benzodiazepines	22 (19.0)	32 (20.8)	49 (49.0)	48 (44.4)	<0.0001
Sleep aids	26 (22.4)	48 (31.2)	30 (30.0)	39 (36.1)	0.16
Total number of medications, mean (±SD)	1.9 (1.6)	2.5 (1.9)	3.1 (2.1)	3.4 (2.0)	<0.0001
Use of one or more symptom-modifying medication	92 (79.3)	131 (85.1)	89 (89.0)	100 (92.6)	0.0272

^a^ *N* = 478 total external sample. Data are *n* (%). BMI, Body mass index; FIQ, Fibromyalgia Impact Questionnaire; FM, Fibromyalgia; SF-36, Medical Outcomes Study 36-item Short Form survey; SNRI, Serotonin norepinephrine reuptake inhibitor; SS, Symptom severity (SS); SSRI, Selective serotonin reuptake inhibitor; TCA, Tricyclic antidepressant; WPI, Widespread pain index. ^b^Low symptom intensity. ^c^Moderate symptoms, low negative mood. ^d^Moderate symptoms, higher negative mood and dyscognition. ^e^High symptom intensity. ^f^ *P*-values are from the omnibus analysis of variance test across the four clusters. . ^g^Lower scores are worse for these scales. ^h^Includes other antidepressants, such as lithium, monoamine oxidase inhibitors, antipsychotics, bupropion, buspirone, lamotrigine and tetracyclic antidepressants.

The percentage of patients on work disability also differed significantly between clusters (*P* <0.0001). Cluster 1 had the lowest percentage of patients on work disability (9.6%), clusters 2 and 3 had a greater number of patients on work disability (18.6% and 19.1% of patients, respectively) and cluster 4 had the greatest percentage of patients on work disability (33.1%).

Medication use across clusters is described in Table 6. In regard to serotonin norepinephrine reuptake inhibitors (SNRIs) and α_2_δ ligands, classes that include the US Food and Drug Administration–approved medications for FM, cluster 4 had the highest percentage of patients taking both SNRIs (44%) and α_2_δ ligands (22%), in comparison to cluster 1, which had 30% of patients receiving SNRIs and 20% taking α_2_δ ligands. Opioid use was significantly different across clusters, with 53% of patients in cluster 4 using opioids in comparison with 18% in cluster 1. Similarly, use of benzodiazepines was different across clusters, with 49% of patients in cluster 3 and 44% in cluster 4 taking benzodiazepines, in comparison to 19% of patients in cluster 1 and 21% in cluster 2.

## Discussion

In the exploratory cluster analysis, we identified four unique clusters using self-report questionnaires representing eight core OMERACT symptom domains (fatigue, sleep disturbance, pain, function, stiffness, dyscognition, depression and anxiety). The four subgroups that were derived from this cluster analysis included (1) a generally low symptom intensity group (cluster 1); (2) a moderate symptom, low anxiety and low depression group (cluster 2); (3) a moderate symptom, higher anxiety and higher depression group (cluster 3); and (4) a generally high symptom intensity group (cluster 4). Despite the fact that the validation sample consisted of younger patients with higher FM symptom severity, the cluster solution in this sample was similar to the exploratory cluster analysis.

Our results share common themes with previous cluster analysis studies in patients with FM. For example, in the cluster analyses by Wilson *et al*. [10], Loevinger *et al*. [7] and de Souza *et al*. [5], there appear to be at least one subgroup of patients who have high physical and psychological symptoms (similar to our cluster 4) and one subgroup with less psychological distress and low levels of physical symptoms (similar to our cluster 1). Notably, our results are quite similar to those of Wilson *et al*. in that we also identified four clusters: one cluster that was low on all symptom domains, two clusters that were moderate on physical domains but distinguished by differences on psychological factors, and one cluster that was high on all symptom domains [10]. Although clusters 2 and 3 had overall moderate levels of symptoms, the severity of depression and anxiety was the distinguishing factor, which is consistent with the findings reported by Loevinger *et al*. [7] and de Souza *et al*. [5] in that psychological distress may be one factor that differentiates clusters.

Our preliminary cluster solution is further supported by FIQ-R and SF-36 scores, which serve as external benchmarks. Cluster 1 in both samples had the lowest mean FIQ-R total score, which also fell within the mild symptom severity range described for the FIQ-R (score 0 to <39) [55]. Compared to this finding, cluster 4 fell in the severe symptom severity range for the FIQ-R (≥59 to 100). Similarly, cluster 1 in both samples had the best physical and mental composite scores on the SF-36, and score ranges appeared relatively close to healthy population norms (50 ± 10) [21]. Cluster 4 in both samples had the worst physical and mental composite scores, indicating the poorest levels of physical and mental health. Using disability status as yet another external benchmark of symptom burden and/or impact, we found that the percentage of patients on work disability significantly increased (*P* <0.0001) as symptom severity increased across clusters.

We were not able to compare medication use across clusters in our exploratory sample (as medication use at the time of the survey was not available). When we compared medication use in the validation sample, however, cluster 1 had the lowest percentage using any medications for FM and cluster 4 had the highest percentage. Although this finding was somewhat surprising, it demonstrates that patients in cluster 4, despite being on multiple medications (patients in cluster 4 were taking an average of 3.4 medications each), had the highest level of symptom burden. This indicates that symptom severity in cluster 4 is not driven by the lack treatment, but rather it may be indicative of the symptom severity spectrum of FM.

In contrast to clusters 1 and 4, clusters 2 and 3 (both exploratory and validation samples) had similar levels of total FIQ-R symptom severity. The cluster differentiation between clusters 2 and 3 was driven primarily by anxiety and depression, with cluster 3 having significantly higher levels. Comparison of medication use in the validation sample indicated that there were no significant differences in medication use between clusters 2 and 3, except with regard to use of benzodiazepines, which was significantly higher in cluster 3 (*P* <0.0001). This indicates that the differences in depression and anxiety that distinguished clusters 2 and 3 were not due to one cluster being differentially treated. In fact, patients in cluster 3 were taking more medications for anxiety (benzodiazepines) compared to cluster 2 and had more severe anxiety.

In support of these results, the SF-36 Mental scores indicated better mental health in cluster 2 compared to cluster 3 in both samples. However, the SF-36 Physical scores were higher in cluster 2 compared to cluster 3 in both samples. A reason for this might be that the increased BMI in cluster 2 could be associated with poorer physical function. Although BMI was significantly higher in cluster 2 in the exploratory sample, the difference in the validation sample did not reach statistical significance.

We recognize several limitations to this study. First, despite identification of four clusters, the sample is cross-sectional and the longitudinal stability of these clusters is unknown. One published study on the longitudinal behavior of FM symptoms over the course of 5.5 years indicated that pain, fatigue and sleep demonstrated small longitudinal fluctuations over time at the group level, but showed significant within-patient variability [56]. A second limitation is that objective biomarkers recommended by OMERACT, such as tenderness, CSF biomarkers and functional imaging, were not available and therefore could not be included in the clustering. A third limitation is that, based on our inclusion criteria, we cannot comment on the generalizability of our results to community samples or men with FM, given our sample of female patients with FM identified through a clinical registry. Fourth, variables such as those included by Docampo *et al*. (sociodemographic characteristics, medications, comorbidities and personal or family history) that we were unable to include [11], and our questionnaire selection could influence clustering; however, the rationale for our use of these variables was based on the OMERACT recommendations, and care was taken to avoid any overlap between items representing symptom domains. Despite this, both our results and those of Docampo *et al*. suggest that varying symptom combinations are present in a heterogeneous sample of patients with FM.

## Conclusions

Our study corroborates previous cluster reports, but also distinguishes itself by being the first study of its kind to incorporate a comprehensive spectrum of symptoms, as recommended by the OMERACT FM working group. We acknowledge that this cluster solution needs longitudinal study and that the feasibility and outcomes of this approach must be assessed before any conclusions regarding its clinical implications can be made; however, the present study is a first step toward this goal.

## Abbreviations

BMI: Body mass index
BPI: Brief Pain Inventory
FIQ-R: Revised Fibromyalgia Impact Questionnaire
FM: Fibromyalgia
GAD-7: Generalized Anxiety Disorder 7-item scale
MASQ: Multiple Ability Self-report Questionnaire
MFI: Multidimensional Fatigue Inventory
MOS-Sleep: Medical Outcomes Study Sleep Scale
OMERACT: Outcome Measures in Rheumatology
PHQ-9: Patient Health Questionnaire
POMS: Profile of Mood States
SF-36: 36-item Short Form survey
SNRI: Serotonin norepinephrine reuptake inhibitor
SS: Symptom severity
SSRI: Selective serotonin reuptake inhibitor
WPI: Widespread Pain Index
